# Estimating freshness of ice storage rainbow trout using bioelectrical impedance analysis

**DOI:** 10.1002/fsn3.1974

**Published:** 2020-11-14

**Authors:** Xinru Fan, Xiaoyu Lin, Chunhua Wu, Nana Zhang, Qiaofen Cheng, Hang Qi, Kunihiko Konno, Xiuping Dong

**Affiliations:** ^1^ National Engineering Research Center of Seafood Collaborative Innovation Center of Provincial and Ministerial Co‐construction for Seafood Deep Processing Liaoning Province Collaborative Innovation Center for Marine Food Deep Processing School of Food Science and Technology Dalian Polytechnic University Dalian 116034 China; ^2^ Department of Food and Nutritional Sciences University of Reading Reading UK

**Keywords:** freshness, hardness, impedance, K‐value, rigor mortis

## Abstract

This study aimed to evaluate the freshness of ice stored rainbow trout by bioelectrical impedance measurements. Rigor mortis, ATP‐related components, K‐value, and hardness of rainbow trout muscle during storage were monitored along with impedance. The results showed that the progress of rigor mortis was accompanied by an increase in impedance. Impedance kept decreasing even in rigor state, and during the gradual resolution of rigor mortis with impedance change upon storage of fish was biphasic (*r* = −0.944, *p* < .01). Thus, when impedance decreased close to the lowest value, K‐value was only about 61.57 ± 0.52%, but still exhibited a high pertinence (*r* = −0.959, *p* < .01). A gradual decrease of the hardness of fish muscle upon storage of fish showed a close correlation (*r* = 0.981, *p* < .01) with impedance decrease. These results suggested that the impedance measurement has a great potential for predicting the freshness of the rainbow trout during ice storage.

## Graphical abstract

This study aimed to evaluate the freshness of ice stored rainbow trout by bioelectrical impedance measurements. These results suggested that impedance parameter could be applied to predict the rainbow trout freshness during ice storage.

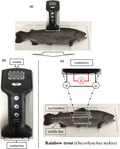

## 1 INTRODUCTION

Owing to the demand for healthy food, such as sashimi and sushi, the global consumption of fresh fish has dramatically increased. Furthermore, consumers are increasingly concerned about the quality of raw fish products. Freshness is one of the most important factors that determine fish quality. Many conventional methods are available to detect fish freshness have been reported. Sensory methods are convenient and quite sensitive to evaluate the fish freshness on‐site, including checking the fading red gill color, loss of eyeball translucency, breaking of belly skin, and development of a bad spoilage smell, etc. (Dutta et al., 2016; Morsy et al., 2016; Sant’ Ana et al., 2011; Sun et al., 2018). Assessment of rigor mortis offers a suitable method to show fish freshness at early stages of storage, and fish in prerigor or full rigor are considered as very fresh. After death, rigor mortis is triggered by the complete consumption of 5' adenosine triphosphate (ATP) in muscle tissue, and the rigor status can be determined simply by placing the fish on the table and observing tail drooping (Bito et al., 1983). The K‐value, which has been well accepted as a freshness index for fish consumed as sashimi products, was also proposed to indicate fish freshness (Uchiyama & Ehira, 1970; Uchiyama et al., 1966). K‐value is calculated by the ratio of ATP‐related compounds, which is associated with rigor mortis (Yoshioka et al., 2019). However, these methods have a request for technical personnel; fish species and stored temperatures are also influential factors. This may lead to confusing freshness assessment.

There are many newly developed rapid and nondestructive methods to determine fish freshness, including near infrared reflectance spectroscopy (NIRS) (Ding et al., 2014), hyperspectral imaging (Khojastehnazhand et al., 2014), computer vision (Dutta et al., 2016; Tinacci et al., 2018), biosensors (Chen, et al., 2017), and electrochemical impedance spectroscopy (EIS) (Sun et al., 2017) which could be used as a rapid detection system in combination with traditional methods. Most of these advanced methods need expensive equipment and specific operating environments, which greatly affect their application in commercial environments or in the field. However, the EIS method might be relatively inexpensive and easy to carry out in a commercial environment; hence, this technology has attracted considerable attention. Owing to rapid changes in proteins and cell structure postmortem, the electrical properties of fish tissue have been found to be closely related to freshness (Sun et al., 2017). It has reported that EIS could be used to judge total volatile base nitrogen (TVB‐N) of fish including carp, sea bream, and squid (Pérez‐Esteve et al., 2014; Sun et al., 2018; Zavadlav et al., 2016). Furthermore, Yuan et al. (2018) proposed that bio‐impedance analysis had a good relationship with the K‐value after 24 hr ice storage. For the ready‐to‐eat raw materials, the textural properties and taste are as important as food safety. However, previous researches mainly focused on the fish shelf‐life rather than fish‐eating quality.

It is well recognized that fishes are considerably more fragile than land animals, such as beef, pork, and lamb, and have a much quicker softening process, which negatively affects meat quality (Morton et al., 2018; Tinacci et al., 2018). Usually, soft fish is often rejected for processing sashimi products. The softening process begins instantly after death and continues until fish muscle is inedible, and it is a complicated biochemical process affected by many factors, including endogenous muscle proteases, and connective tissue and sarcoplasmic protein degradation (Ahmed et al., 2015). This phenomenon is much severer in salmon industry, salmonids lose their hardness within a much shorter period compared with cyprinids, especially in early phase after postmortem. Besides, the hardness of fish muscle only can be detected by destructive methods (Gaardera et al., 2012; Li et al., 2017). Therefore, how to identify the fish muscle quality of salmonids without damage is an urgent thing.

In the present study, it started after fish immediately slaughter, and the early storage phase was investigated particularly. The changes during the early storage period after fish body death were investigated. The feasibility of using impedance measurements to determine the freshness of fish during storage was evaluated by exploring the relationship between conventional freshness indices and impedance properties of fish. The freshness measurements included rigor mortis, ATP decomposition, K‐value, and hardness of the samples. This method could be used nondestructively to determine fish freshness with high efficiency.

## 2 MATERIAL AND METHODS

### 2.1 Fish

Rainbow trout (*Oncorhynchus mykiss*, *n* = 18, weight 800 ± 50 g, length 43 ± 1 cm) in a water tank at around 10°C were purchased alive from a local market. The fish were killed at the market and brought to the laboratory on ice within 30 min. After arrival at the laboratory, the fish were stored in iced water, and changes of the biochemistry and physical properties were measured immediately. The body temperature of the fish was at 3–4°C when the experiment began and usually dropped to 0°C in approximately 1 hr.

### 2.2 Rigor mortis (RM) measurement

The onset and progression of rigor mortis were measured according to the simplified method reported by Bito et al. (1983). A half‐length of the fish body (containing the head region) was placed on the table, and the tail region freely drooped. The vertical distance (cm) from the center of the body to the tail end was measured as the rigor mortis index and more simply expressed. The procedure was carried out at room temperature of 20°C within 1 min to avoid the fish body temperature rise. When the fish body showed full postrigor, the test was finished. Three fish similar in size were used in this experiment, and then, they were tested at 0–36 hr (interval 2 hr) and 36–72 hr (interval 4 hr) theses points.

### 2.3 Analysis of ATP decomposition processes and K‐value calculation

Adenosine triphosphate and its decomposed compounds, including adenosine diphosphate (ADP), adenosine monophosphate (AMP), inosine 5'‐monophosphate (IMP), hypoxanthine (Hx), and inosine **(**HxR), were extracted from fish muscle using a simplified method of Hu et al. (2013). The same fish was used after rigor mortis and was measured. Small muscle pieces (1.0 g) were taken from the dorsal part of the fish body near its head at 0, 1, 3, 5, 9, 13–125 hr (interval 4 hr) during the whole storage period, and ATP and its decomposed compound contents of the muscle were measured with three replicates. The region for taking a small piece of muscle sample was carefully selected to avoid affecting the fish rigor mortis measurements. Briefly, 1.0 g of fish muscle was mixed with 10 ml of 5% perchloric acid and kept for 15 min on ice. Then, 5.2 ml of 1 M KOH and 4.8 ml of H_2_O were added to the mixture to adjust the mixture solution to pH 2.5–3. The supernatant obtained after standing on ice for 15 min was then filtered using a membrane (0.22 μm, Tianjin Branch billion Lung Experiment Equipment Co., ltd.). ATP and its degraded compounds were analyzed using a Shimadzu LC‐20AD HPLC system (Shimadzu) with COSMOSIL 5C18‐PAQ Packed column (4.6 ID × 250 mm, NACALAI TESQUE, INC.) (Li et al., 2017). The compounds were eluted with 0.1 M phosphate buffer solution at pH 3 and detected at a 254 nm wavelength. The standard curve was obtained by mixed components, and each concentration of standard was 100.0 μg/ml.

The K‐value of the muscle was calculated based on the contents of ATP and its degraded compounds and presented as the relative amounts of the sum of HxR and Hx to the total amount of compounds coming from ATP (in percent) (Uchiyama & Kakuda, 1984; Uchiyama et al., 1966). The equation is shown as follows:

K‐value (%) = $\frac{\left(\mathrm{HxR}+\mathrm{Hx}\right)}{\left(\mathrm{ATP}+\mathrm{ADP}+\mathrm{AMP}+\mathrm{IMP}+\mathrm{HxR}+\mathrm{Hx}\right)}\times 100$

### 2.4 Muscle hardness

Cuboid muscle with a dimension of 25 mm × 20 mm × 10 mm was excised from the dorsal part of the fish. A TA‐XT Plus Texture Analyzer (Stable Micro Systems Ltd.) was used to measure the hardness with the following setting: Load cell 5 kg, 10 mm diameter spherical probe (P/0.5S), probe speed at 0.5 mm/s. The sample was placed on the platform with the skin side down, while the hardness was measured on the flesh side of the sample. Three samples were measured, and the average was used to represent the hardness of the sample. Samples were taken at 0 hr, 1 hr, 3 hr, 5 hr, after that every 4 hr after the fish was killed until the fish muscle was too soft to complete the test.

### 2.5 Impedance measurement

**FIGURE 1:**
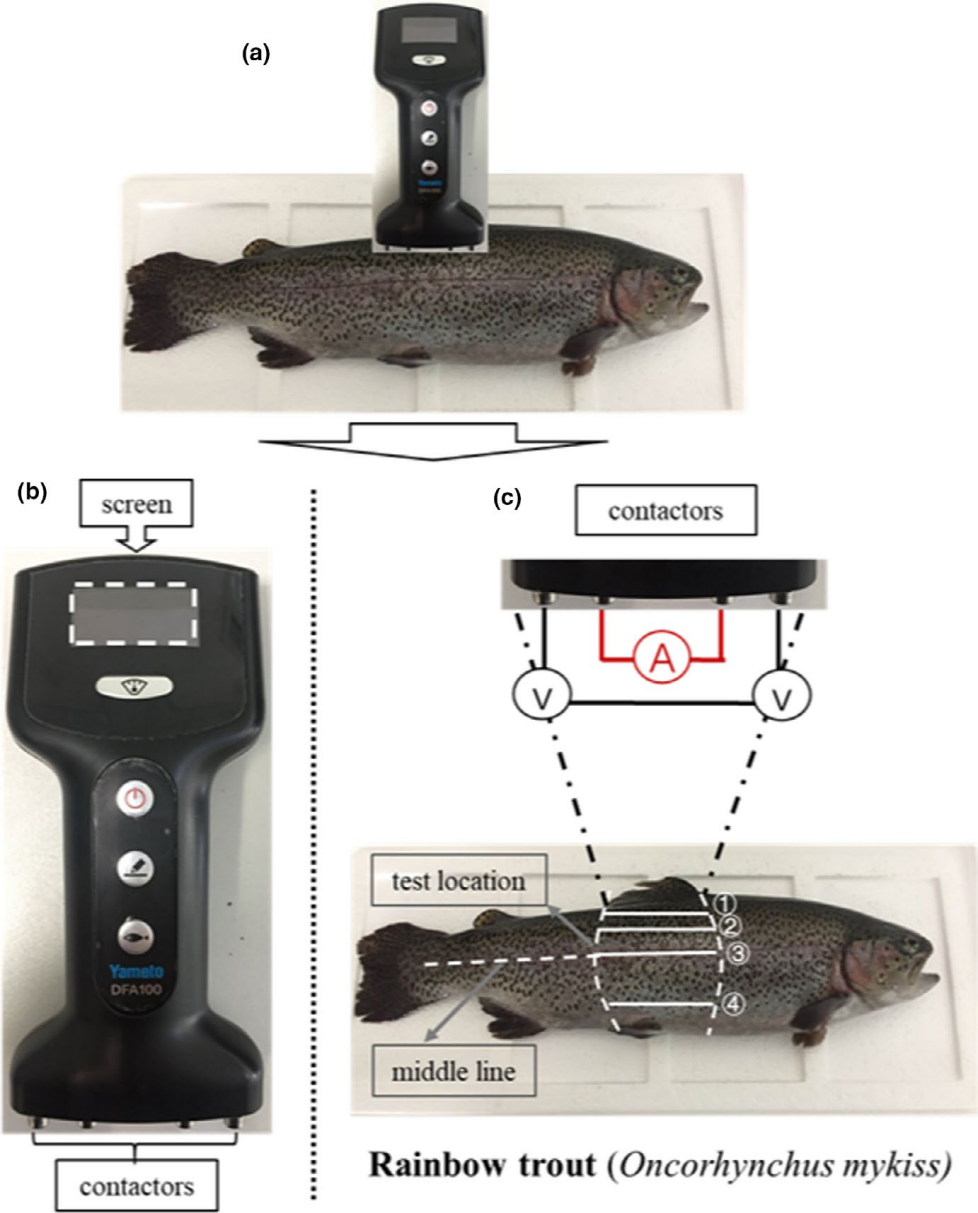
The procedure of impedance measurement. (a) The method of impedance measurement; (b) the instrument for impedance measurement, which contents two major parts: screen and contactors; (c) the principle of the instrument, and ①, ②, ③and ④ fixed test locations are showed. A, current; V, voltage

Fish impedances were measured using a model DFA100 Fish Analyzer^TM^ (Yamato Scale Co Ltd), which can produce five different frequencies at 2, 5, 10, 20, and 100 kHz (Figure 1a). The instrument had four contactors, and all of them can touch the surface of fish skin, so they form a complete circuit, and resistance (impedance) of the fish body was displayed on the screen (Figure 1b). Impedance was measured at four fixed positions of the fish body, marked ①, ②, ③, and ④ in Figure 1c. The distance from the dorsal fin to ①–④ was 1–1.5 cm (back 1), 3 cm (back 2), 5.5 cm (middle), and 9 cm (belly), respectively. The measurements were taken at 0, 1, 3, 5, 9, and 13–125 hr with 4 hr intervals or a multiple of 4 hr during the whole storage period and performed in triplicates.

### 2.6 Statistical analysis

Results were presented as mean values ± standard deviation (*SD*). Data were analyzed using SPSS software (SPSS 17.0 for Windows, SPSS Inc.), and analysis of variance (one‐way ANOVA) was used to determine the difference between samples at a significance level of 0.05. For a significant difference, Turkey's multiple comparison test was used to investigate the difference between samples. Linear regression was used to explore the relationship between variables, including impedance, K‐value, rigor mortis index, and hardness.

## 3 RESULTS AND DISCUSSION

### 3.1 Change in impedance of fish during storage

**FIGURE 2:**
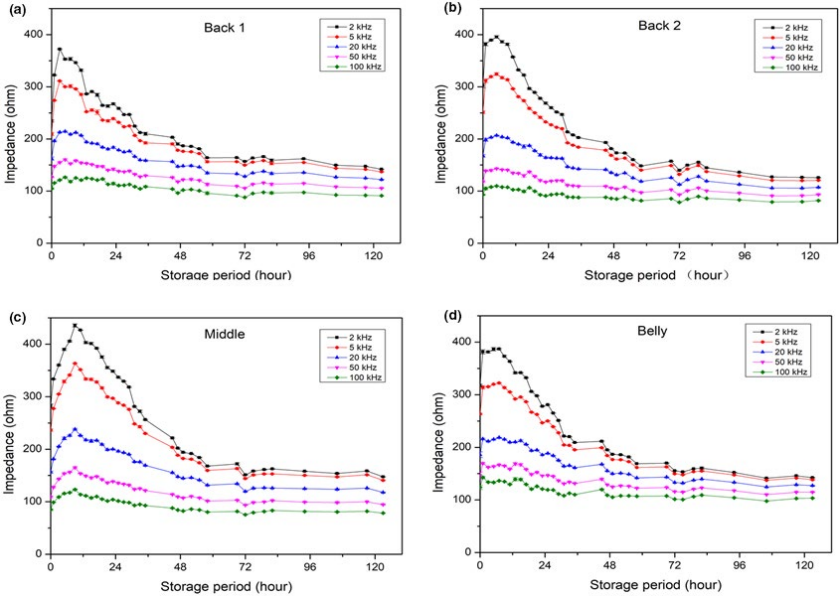
Changes of impedance during ice storage of rainbow trout were measured at various frequencies and different positions. From (a) to (d) represents, respectively, the four different positions (①–④ were showed in Figure 1) effect on impedance at five different frequencies including 2 kHz, 5 kHz,20 kHz,50 kHz, and 100 kHz, respectively

Changes in the impedance of the fish measured at different positions and frequencies are presented in Figure 2. The impedance at the belly portion was the highest (317.50 ± 2.22 Ohm), and the back 1 portion showed the lowest value (234.29 ± 1.90 Ohm) among the four selected measuring locations at the beginning. The impedance variation could be explained by the lipid content. The belly portion of fish muscle usually contains more lipid than the dorsal part, which could increase fish impedance. Similar results have been reported by Li et al. (2019); Pomfret had higher fat content than hairtail but showed higher impedance than hairtail. Changes in the impedance during storage were slightly different among the four locations. The increase of impedance at the belly was the smallest with an increase ratio of 1.2 in 3 hr of storage as compared with all other positions. However, the time needed for a peak value measured in 3 hr at the belly was the same as that measured at dorsal muscle positions. Measurement at the middle part of the fish body showed a large increase in impedance, which was 1.56 times higher than the initial values in 9 hr. Beneath the skin at the middle position, the surface dark muscle has high lipid and myoglobin contents, which could partly explain the impedance variations. However, more investigation is still needed to understand the mechanism of this phenomenon. According to the above results, the dorsal muscle position gave the most sensitive results than other positions. This is reasonable because the dorsal part is targeted in fishes to study its quality or its freshness.

As expected, 2 kHz gave the highest impedance among the five frequencies for any samples, whereas 100 kHz gave the lowest reading. The initial impedance for the fish at 2 kHz was about 234.29 ± 1.90 Ohm, whereas that at 100 kHz was only 104.74 ± 1.65 Ohm. The maximum impedance at 2 kHz (372.26 ± 1.14 Ohm) was obtained in 3–5 hr of storage, and increased by roughly 1.58 times, whereas the increase at 100 kHz was only 1.13 times. The impedance decreased gradually with further fish storage up to 125 hr (5 day). The value for the fish stored for 5 day was 148 and 98 Ohm at 2 and 100 kHz, respectively (Figure 2a). The difference in the impedance between the two frequencies became smaller than that at the initial stage. The ratio of impedance between 2 and 100 kHz was 2.28 at the start, whereas it decreased to 1.51 after 5 day of storage. This indicated that alternating current does not travel easily through fresh fish but does in stored fish. It is possible because the breakdown of muscle tissue membrane promotes current penetration even though postmortem fish is fresh. The much severer phenomenon of frozen‐thawed sea bream impedance properties was explained by Fuentes et al. (2013), where free electrolytes were released in tissue when cell membranes were broken to increase its conductivity, resulting in decreased impedance. Changes in the impedance measured at different frequencies showed a similar trend, that is, biphasic, increasing initially and decreasing in later phases. Therefore, measuring at a frequency of 2 kHz 1–1.5 cm below the dorsal fin could provide more sensitive impedance reading, and data collected at 2 kHz were used for further comparison and correlation analysis. The quantification model was applied to impedance results, based on Multifarious Partial Least Squares (Ding et al., 2014), the equation was y = 0.0263x^2^‐4.8657x + 366.02 (y: impedance, x: storage time, *r* ^2^ = 0.9603) for “2kHz, 1–1.5cm” testing condition.

### 3.2 Rigor mortis and ATP decomposition during fish storage

**FIGURE 3:**
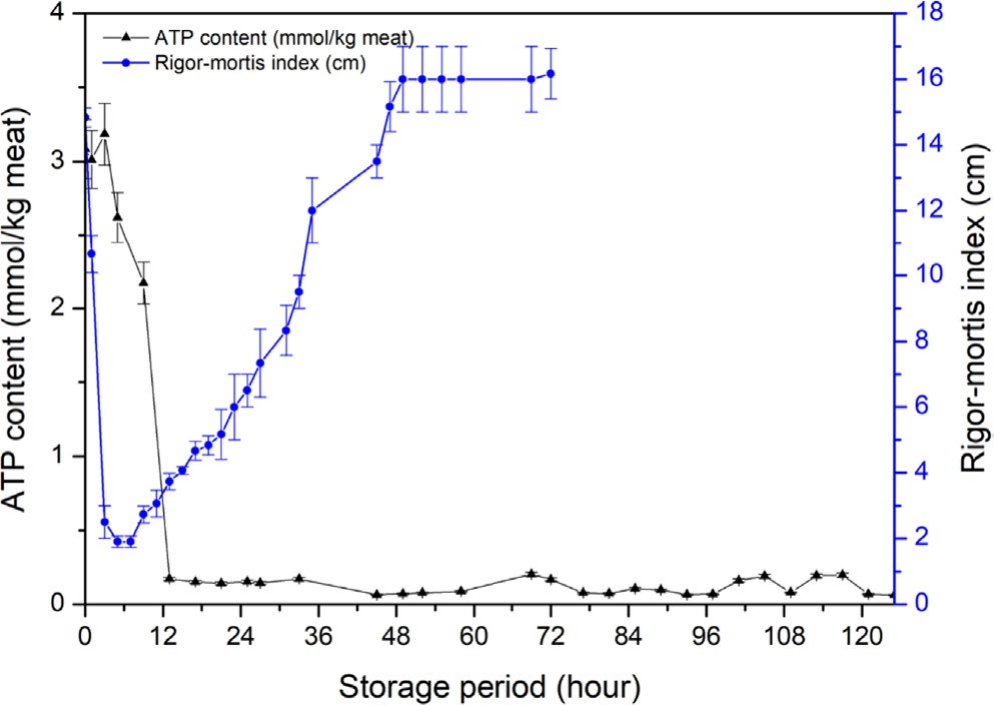
Decomposition of ATP and rigor mortis onset and resolution were showed during ice storage

Rigor mortis changes and ATP decomposition were measured immediately when samples arrived at the laboratory, and the results are shown in Figure 3. Because of different types of fish muscles (mainly smooth muscles and sinews) contracted when fish postmortem, the appearance of fish body can be used to identify rigor status (Amlacher, 1961). Rigor mortis development was mainly represented by the vertical distance of tail drooping (Bito et al., 1983). Usually, glycolysis occurs when no oxygen enters the muscle cells of the slaughtered fish, resulting in a >60% creatine phosphate loss and no ATP reforming. Eventually, the calcium ions cannot be transferred because of no supporting energy, leading to muscle contraction (Hultin, 1984). The fish gradually turned to rigor mortis in only 3 hr of storage, the RM index decreased from 14.83 ± 0.29 to 2.50 ± 0.50 cm. Full rigor mortis was reached at about 5 hr showing 1.90 ± 0.17 cm, and the rigidness of the fish continued to increase to approximately 9 hr of storage. The rigidness then started to decline gradually with further storage of the fish, and a complete resolving of rigor mortis was obtained at 48 hr of storage. Similar results of sea‐farmed rainbow trout and Atlantic salmon have been reported based on the crowding and pumping procedures of preslaughter samples status and stunning methods; therefore, rigor mortis were detected in different death time (3 to 12 hr) and accompanied by a different reduction in rigidness (Merkin et al., 2010, 2014; Roth et al., 2012).

The fish contained 3.08 ± 0.20 mmol/kg of ATP immediately after delivery to the laboratory. However, ATP in the fish was considerably degraded after death as the muscle contained certain 1.29 ± 0.26 mmol/kg of ADP and a relatively large IMP concentration (2.62 ± 0.14 mmol/kg). Usually, approximate 10 mmol/kg was contained in alive fish muscle to maintain normal physical activities (Yoshioka et al., 2019). After fish dead, there is no oxygen supply causing no reproduce the ATP from ADP in fish muscle, simultaneously rigor status occurred (Iwamoto et al., 1990). ATP hydrolysis was related to enzyme reactions, containing ATP to ADP (ATPase), AMP to IMP (AMP deaminase), and IMP to HxR (5′‐nucleotidase) (Wei et al., 2020). In present study, approximately 70% of ATP in the muscle had been degraded during transportation to the laboratory (Table 1). The ATP content decreased to zero near 12 hr of storage, which was much later than the occurrence of rigor mortis. The result clearly showed that ATP degradation into ADP and ADP decomposition into IMP in the rainbow trout muscle was relatively fast that meant the ATPase and AMP deaminase activities were quite positive after slaughter. In addition, both various fish species and muscle cell sizes affect rigor mortis processing (Lerfall et al., 2017). Furthermore, some fish breeding conditions containing crowding density and pumping conditions before slaughter, and stunning methods contributed to the acceleration or delay of the onset of rigor mortis (Merkin et al., 2010; Roth et al., 2012).

**TABLE 1:** Adenosine triphosphate and its related compounds were analyzed during the ice storage

Time (hour)	ATP (mmol/kg)	ADP (mmol/kg)	AMP (mmol/kg)	IMP (mmol/kg)	HxR (mmol/kg)	Hx (mmol/kg)
0	3.083 ± 0.201^de^	1.292 ± 0.259^b^	0.443 ± 0.08^b^	2.620 ± 0.135^ab^	0.170 ± 0.016^a^	0.115 ± 0.005^a^
1	3.011 ± 0.196^d^	1.225 ± 0.246^b^	0.342 ± 0.080^ab^	2.478 ± 0.128^a^	0.177 ± 0.017^a^	0.112 ± 0.005^a^
3	3.184 ± 0.208^e^	1.230 ± 0.247^b^	0.312 ± 0.073^ab^	2.469 ± 0.137^ab^	0.289 ± 0.027^a^	0.116 ± 0.005^a^
5	2.620 ± 0.171^c^	1.186 ± 0.238^b^	0.306 ± 0.072^ab^	2.566 ± 0.133^ab^	0.255 ± 0.024^a^	0.112 ± 0.005^a^
9	2.171 ± 0.142^b^	1.159 ± 0.232^b^	0.288 ± 0.068^ab^	2.368 ± 0.122^a^	0.340 ± 0.032^a^	0.117 ± 0.005^a^
13	0.171 ± 0.011^a^	0.659 ± 0.132^a^	0.259 ± 0.061^ab^	5.382 ± 0.278^m^	0.887 ± 0.083^a^	0.115 ± 0.005^a^
17	0.151 ± 0.010^a^	0.704 ± 0.141^a^	0.244 ± 0.057^ab^	5.177 ± 0.268^lm^	0.868 ± 0.081^a^	0.115 ± 0.005^a^
21	0.141 ± 0.009^a^	0.707 ± 0.142^a^	0.280 ± 0.066^ab^	5.420 ± 0.280^m^	0.988 ± 0.092^a^	0.135 ± 0.006^a^
25	0.154 ± 0.010^a^	0.686 ± 0.138^a^	0.266 ± 0.062^ab^	5.430 ± 0.281^m^	2.555 ± 0.239^b^	0.182 ± 0.008^ab^
29	0.145 ± 0.009^a^	0.565 ± 0.113^a^	0.281 ± 0.066^ab^	4.938 ± 0.255^kl^	2.813 ± 0.263^b^	0.194 ± 0.008^b^
33	0.171 ± 0.011^a^	0.534 ± 0.107^a^	0.263 ± 0.062^ab^	4.635 ± 0.240^ijk^	3.822 ± 0.358^cd^	0.287 ± 0.012^c^
37	0.180 ± 0.012^a^	0.462 ± 0.093^a^	0.241 ± 0.057^ab^	4.815 ± 0.249^kl^	3.744 ± 0.350^cd^	0.273 ± 0.011^c^
41	0.082 ± 0.005^a^	0.484 ± 0.097^a^	0.242 ± 0.057^ab^	4.706 ± 0.243^jk^	3.589 ± 0.336^c^	0.306 ± 0.013^c^
45	0.065 ± 0.004^a^	0.464 ± 0.093^a^	0.235 ± 0.055^a^	4.666 ± 0.241^jk^	3.794 ± 0.354^cd^	0.297 ± 0.012^c^
49	0.072 ± 0.005^a^	0.481 ± 0.096^a^	0.240 ± 0.056^ab^	4.173 ± 0.216^fgh^	3.699 ± 0.346^cd^	0.383 ± 0.016^d^
53	0.077 ± 0.005^a^	0.475 ± 0.095^a^	0.243 ± 0.057^ab^	4.348 ± 0.225^hij^	3.848 ± 0.360^cd^	0.437 ± 0.018^e^
57	0.089 ± 0.006^a^	0.424 ± 0.085^a^	0.272 ± 0.064^ab^	4.285 ± 0.222^hij^	3.891 ± 0.364^cde^	0.605 ± 0.025^f^
61	0.193 ± 0.013^a^	0.443 ± 0.089^a^	0.263 ± 0.062^ab^	4.314 ± 0.223^hij^	4.156 ± 0.389^cdef^	0.643 ± 0.027^fg^
65	0.158 ± 0.010^a^	0.493 ± 0.099^a^	0.275 ± 0.065^ab^	3.707 ± 0.192^efg^	4.475 ± 0.419^cdef^	0.678 ± 0.028^g^
69	0.203 ± 0.013^a^	0.431 ± 0.086^a^	0.278 ± 0.065^ab^	3.735 ± 0.193 ^efg^	4.595 ± 0.430^cdef^	0.723 ± 0.030^h^
73	0.168 ± 0.011^a^	0.436 ± 0.087^a^	0.288 ± 0.068^ab^	3.534 ± 0.183^def^	4.680 ± 0.438^edf^	0.809 ± 0.033^i^
77	0.078 ± 0.005^a^	0.476 ± 0.096^a^	0.247 ± 0.058^ab^	3.779 ± 0.195 ^efg^	4.560 ± 0.426^cdef^	0.806 ± 0.033^i^
81	0.073 ± 0.005^a^	0.413 ± 0.083^a^	0.255 ± 0.060^ab^	3.313 ± 0.171^cde^	4.308 ± 0.403^cdef^	0.807 ± 0.033^i^
85	0.107 ± 0.007^a^	0.426 ± 0.085^a^	0.266 ± 0.062^ab^	3.528 ± 0.182^def^	4.364 ± 0.408^cdef^	0.826 ± 0.034^i^
89	0.098 ± 0.006^a^	0.459 ± 0.092^a^	0.251 ± 0.059^ab^	3.408 ± 0.176^cde^	4.569 ± 0.427^cdef^	0.910 ± 0.038^j^
93	0.065 ± 0.004^a^	0.426 ± 0.085^a^	0.264 ± 0.062^ab^	3.417 ± 0.177^cde^	4.658 ± 0.436^edf^	0.946 ± 0.039^jk^
97	0.070 ± 0.005^a^	0.481 ± 0.096^a^	0.268 ± 0.063^ab^	3.972 ± 0.205^fgh^	4.324 ± 0.404^cdef^	0.930 ± 0.038^jkl^
101	0.161 + 0.011^a^	0.427 ± 0.086^a^	0.280 ± 0.066^ab^	3.450 ± 0.178^cdef^	4.322 ± 0.404^cdef^	1.038 ± 0.043^m^
105	0.190 ± 0.012^a^	0.461 ± 0.093^a^	0.279 ± 0.065^ab^	3.654 ± 0.189 ^def^	4.691 ± 0.439^def^	0.997 ± 0.041^klm^
109	0.080 ± 0.005^a^	0.444 ± 0.089^a^	0.249 ± 0.059^ab^	3.567 ± 0.184 ^def^	4.899 ± 0.458^ef^	1.005 ± 0.041^lm^
113	0.194 ± 0.013^a^	0.485 ± 0.097^a^	0.282 ± 0.066^ab^	3.495 ± 0.181^cdef^	4.882 ± 0.457^ef^	0.977 ± 0.040^klm^
117	0.198 ± 0.013^a^	0.430 ± 0.086^a^	0.271 ± 0.064^ab^	3.127 ± 0.162^cd^	5.158 ± 0.482^f^	0.968 ± 0.040^jkl^
121	0.068 ± 0.004^a^	0.488 ± 0.098^a^	0.241 ± 0.056^ab^	3.118 ± 0.161^cd^	5.030 ± 0.470^f^	0.946 ± 0.039^jkl^
125	0.063 ± 0.004^a^	0.471 ± 0.094^a^	0.247 ± 0.058^ab^	2.976 ± 0.154^bc^	5.027 ± 0.470^f^	0.993 ± 0.041^klm^

Note

Different letters (a‐m) within each row indicate significant differences (*p* < .05).

Adenosine triphosphate and its derived compounds were analyzed during fish storage as indicated in Table 1. The prominent compound detected at an early storage stage was IMP, showing a maximum content of 5.43 ± 0.28 mmol/kg meat in 25 hr. Its content remained high from 25 to 125 hr (remaining 3.13 ± 0.16 mmol/kg in 125 hr) with a very slight decrease during storage, which suggested that IMP has the slowest decomposition rate among ATP and its decomposition components. In accordance with the decrease of IMP content, HxR content increased. HxR was slowly converted into Hx. Storage of the fish for 125 hr still contained a certain amount of IMP, although progressive degradation of IMP occurred.

**FIGURE 4:**
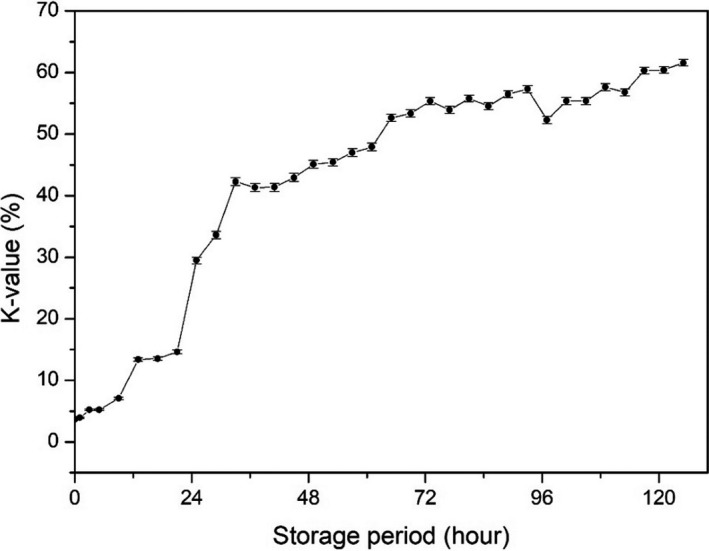
Changes of K‐value during ice storage of rainbow trout

The K‐value was calculated as shown in Figure 4. The initial K‐value was 3.70 ± 0.11% and increased gradually with storage reaching 61.57 ± 0.52% in 125 hr (roughly 5 day). Generally, it has been recognized that a K‐value <10% is acceptable for instantly killed fish, and <20% for top‐grade sushi and sashimi (Yuan et al., 2018). These results were consistent with previously reported K‐values in common carp and chub mackerel starting storage at 9.45% and 8.7%, respectively (Kuda et al., 2008; Li et al., 2017). It was reported that the increasing profile of the K‐value differs between species (Uchiyama et al., 1966). Generally, cold‐water fishes tend to show a quick increase of K‐value, whereas warm water species show a slow increase of K‐value (Uchiyama et al., 1966). The K‐value of red sea bream increased slowly to 30% over 25 day, horse mackerel increased to 40% in 14 day, and flounder increased to 70% within 7 day (Yoshioka et al., 2019; Yuan et al., 2018). The K‐value increase obtained in the present study with rainbow trout, a cold‐water fish species, was a little slower than that of cod and much faster than that of sea bream (Uchiyama et al., 1966).

### 3.3 Correlation between rigor mortis index and impedance change

**FIGURE 5:**
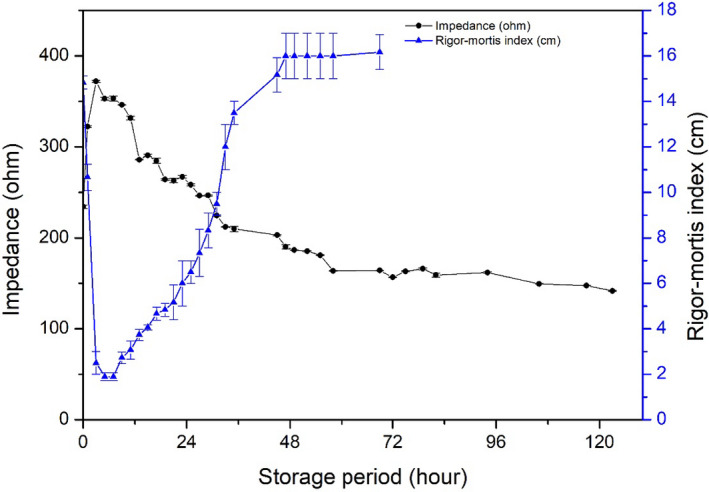
Comparison of impedance change and rigor mortis and its resolution during the storage. Changes in impedance and rigor mortis were compared

Change in impedance measured at 2 kHz and the rigor mortis index are shown in Figure 5. When fish showed full rigor mortis with minimal distance in 2.50 ± 0.50 cm at 3 hr of storage, impedance increased to a maximal value. As the rigor mortis and impedance were measured on the same fish, it was clearly demonstrated that the impedance increase was accompanied by the rigor mortis progression. The fish kept its rigor status for a few hours, up to 7–9 hr of storage. Simultaneously, the impedance began to decrease after reaching the maximal value. Fish required 48 hr to show a full resolution of its rigor state. During this resolution process of rigor, the impedance constantly decreased until rigor mortis was resolved completely at 48 hr of storage. The impedance decrease seemed to stop at approximately 100 hr. Impedance could still be detected, even when rigor mortis was resolved and did not change further.

### 3.4 Correlation between muscle softening and impedance change during fish storage

**FIGURE 6:**
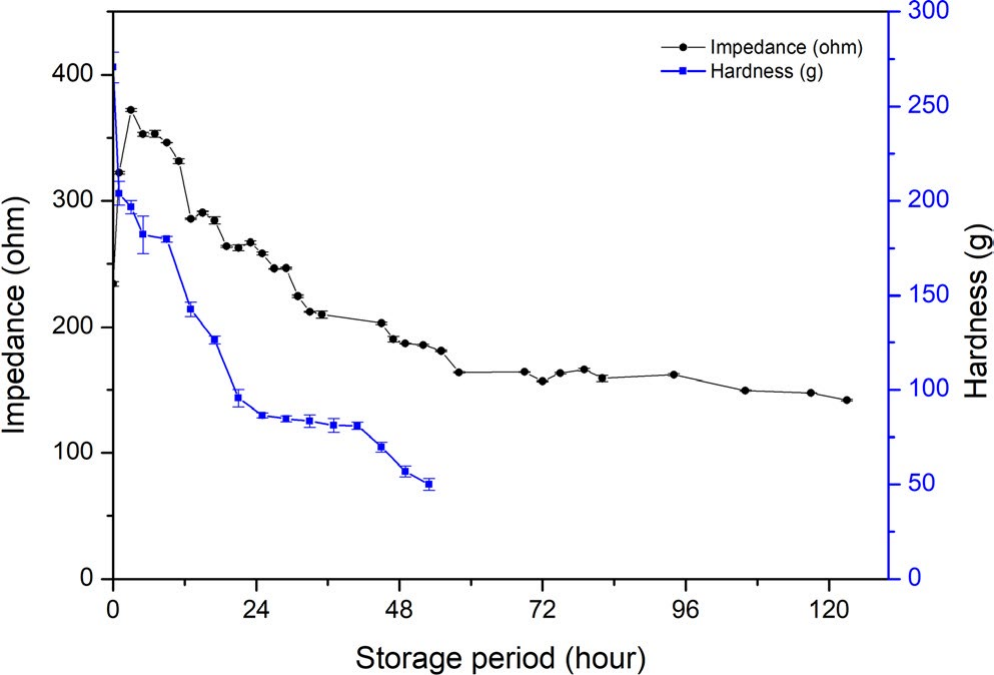
Change in hardness of fish muscle during ice storage. Changes in hardness of fish muscle during the storage of the fish were measured and were compared with that of impedance

Change in the hardness of rainbow trout muscle during its storage is shown in Figure 6. Hardness of the fish muscle was relatively high (270.60 ± 8.13 g) at the start of storage, but the value dropped quickly within 1 hr (204.02 ± 6.21 g), and then, the value decreased constantly to 56.90 ± 2.99 g at 48 hr of storage, to approximately 1/5 of the initial value. The change was monophasic, not biphasic, as found with impedance (Figure 6). In commercial fishing and transportation usually takes a certain amount of time, the increasing phase of impedance only within initial 3 hr in present study, so it is possible to ignore. Once the increasing impedance in the early phase was ignored, impedance decrease and loss of hardness of fish muscle seemed correlated (*r* = 0.666, *p* = .09), as indicated in Table 2. These results demonstrated that the impedance decrease could be used to detect the softening of fish muscle.

**TABLE 2:** Pearson's correlation coefficients between impedance and K‐value, rigor mortis index, and hardness were calculated

	Impedance	K‐value	Rigor mortis index	Hardness
Impedance	1	−0.938**	−0.893**	0.666
K‐value		1	0.745*	−0.883**
Rigor mortis index			1	−0.293
Hardness				1

* *p* < .05.

** *p* < .01.

**FIGURE 7:**
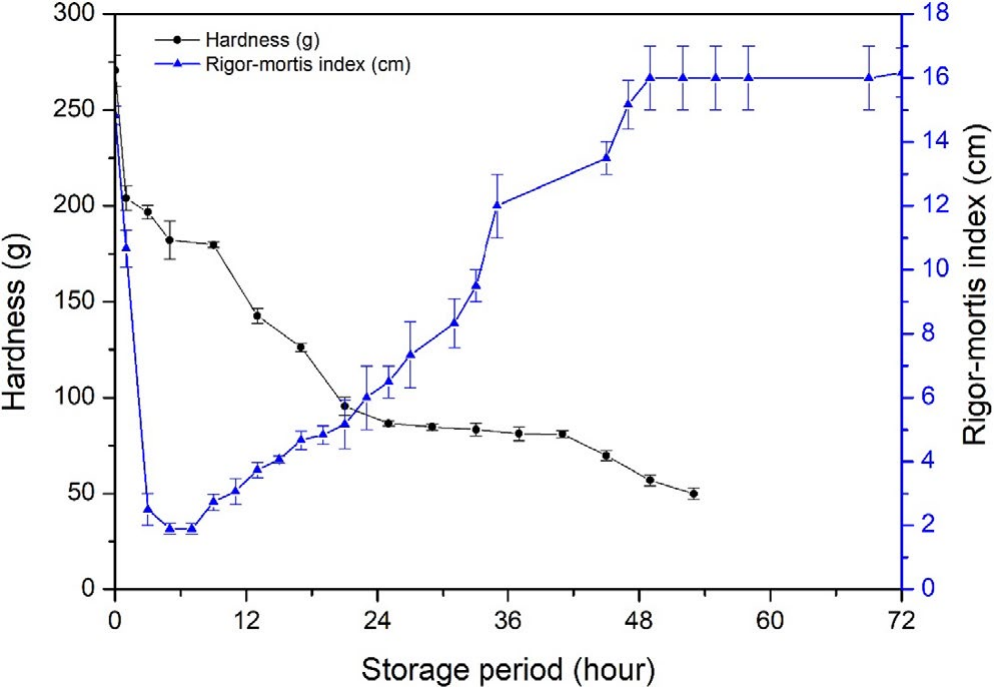
Comparison of rigor mortis and its resolution with hardness of fish muscle. Changes hardness of fish muscle and rigor mortis were compared

It should be noticed that fish muscle turned soft throughout the storage period from the beginning regardless of rigor mortis occurrence (Figure 7). As mentioned before, the initial fish body temperature was 3–4°C when the analysis started, and then it dropped to 0°C within an hour. The hardness decreased from 270.60 ± 8.13 g to 204.02 ± 6.21 g in 1 hr, and decreased to 196.80 ± 3.50 g in 3 hr, 182.20 ± 0.17 g in 5 hr, where fish turned to rigor state in 3 hr and showed minimum RM index in 5 hr. Therefore, the temperature dropped might have effect on fish muscle texture in early phase (within one hour); once fish body temperature kept balance in 0°C, the hardness lost gradually. Besides, the fish muscle in rigor mortis did not show the hardest texture. It seemed that the decrease of hardness was independent with the rigor mortis phenomenon development. In addition, it has reported that fish muscle fibers did not stop losing their regular shape and uniform size when the rigor mortis development (Shi et al., 2020). The muscle microstructure would have been damaged during rigor mortis onset and resolution, because of muscle contraction and release (Nakayama et al., 2017). Besides, when ATP decomposed, the myosin and actin produced a complex formation, which can restrict the flexibility of muscle in order to raising the stiffness (Edgar et al., 2013).

### 3.5 Correlation between K‐value and impedance value

**FIGURE 8:**
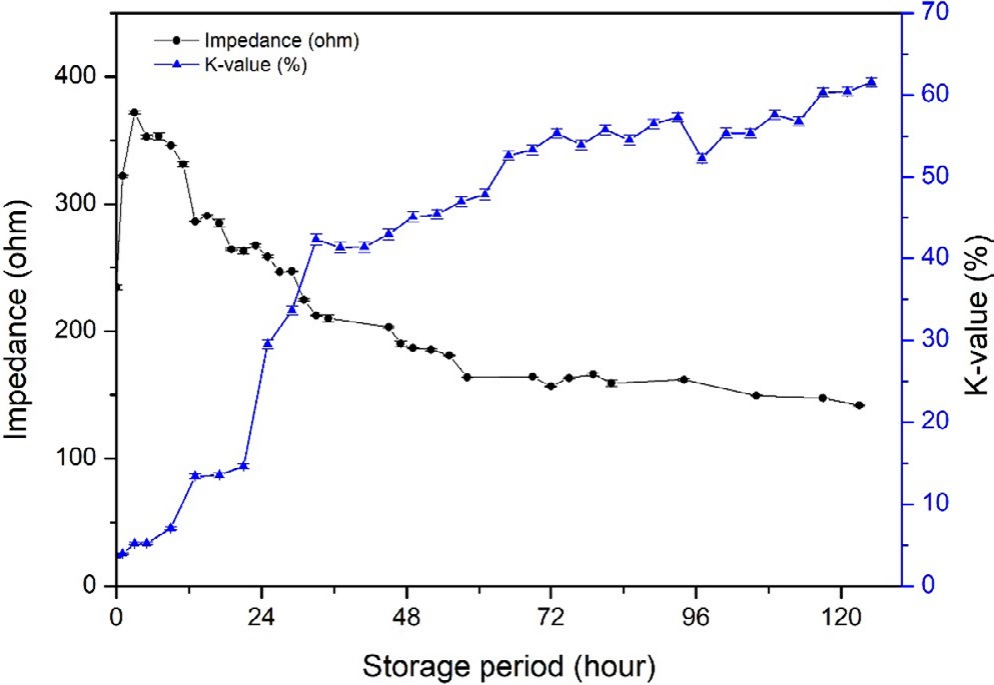
Comparison of K‐value and impedance of fish muscle. Changes impedance of fish meat and K‐value were compared

K‐value is a useful freshness indicator. Changes in impedance and K‐value during the storage were compared (Figure 8). K‐value increased gradually during storage in a monomeric manner, whereas the change in impedance was biphasic, which consisted of an increasing phase in an early period and a decreasing phase in the latter period. However, if the increasing phase in the early storage period was ignored, then two changes were correlated. The K‐value increased steadily up to 72 hr of storage and then stabilized. The K‐value at 125 hr of storage was 61.57 ± 0.52%. Impedance decreased steadily from 3 hr of storage. In this study, the experiment was designed at an ideal state, where the fish were killed and analyzed immediately. However, fish are not usually consumed in such a short time. Therefore, if we ignore the early phase of impedance increasing during transportation, the impedance is easier to measure and can be applied in a commercial environment and can be compared with the K‐value.

K‐value <60% was recognized as commercial fish processing (Yuan et al., 2018), in present result when fish sample stored less 117 hr, the K‐value <60%. Basing on this situation, impedance was 157.92 calculated by the quantification model (y = 0.0263x^2^‐4.8657x + 366.02). The initial increasing phase might confuse the judgment, but the start point impedance value (234.29 ± 1.90 ohm) of was higher than 157.92. Therefore, impedance over 157.92 can be used as evaluated standard for rainbow trout whole period stored on ice.

### 3.6 Correlation between impedance and other indexes

To understand the relationship between impedance and other changes mentioned above, Pearson's correlation analysis was employed to explore their relationship (Table 2). There was a strong negative correlation between impedance and K‐value (*r* = −0.938, *p* < .01) and distance of the tail tip from the table (*r* = −0.893, *p* < .01), and a less positive correlation between impedance and hardness (*r* = 0.666, *p* < .01).

Because impedance and K‐value had a good relationship during the storage period, impedance could be used to detect change of K‐value during the early phase (< 3.70 ± 0.11%), where rigor mortis was accompanied by increased impedance. Once fish passed through the rigor mortis stage or were in the rigor‐resolution stage, a relatively strong negative correlation between K‐value and impedance occurred with *r* = −0.959 (*p* < .01) as shown in Table 3. An increase in K‐value requires the production of HxR or Hx; consequently, the K‐value for fish before rigor mortis was close to 0%. Therefore, it is reasonable to remove this phase (before rigor mortis) from the analysis. Impedance is a relatively good index to predict K‐value for fish reaching rigor mortis. Meanwhile, impedance showed an accepted relationship with hardness (*r* = 0.981, *p* < .01). In this case, impedance would be an available method to measure fish freshness. As the K‐value increase is species‐specific, and there is no information about impedance changes for other fish species, the exact correlation for other fish species is uncertain. Moreover, the K‐value of rainbow trout in 125 hr was 61.57 ± 0.52%, whereas the impedance values were minimal. To expand the application of impedance measurement to predict K‐value for other fish species, more careful studies should be carried out. However, the impedance measurement can potentially be used as a good index to predict K‐value.

**TABLE 3:** Pearson's correlation coefficients between impedance and K‐value, rigor mortis index, and hardness were calculated (without increasing phase, means removing 0 hr and 1 hr)

	Impedance	K‐value	Rigor mortis index	Hardness
Impedance	1	−0.959**	−0.944**	0.981**
K‐value		1	0.873*, **	−0.982**
Rigor mortis index			1	−0.879**
Hardness				1

* *p* < .05.

** *p* < .01.

The correlation coefficient between muscle hardness and its impedance was 0.666 (*p* = .09). A rapid drop in the hardness of fish muscle was found in the early stage of storage before rigor mortis, which may have affected the correlation between the two attributes. If the data before rigor mortis were removed, the decrease in meat hardness correlated well with the decrease of impedance (0.981, *p* < .01, Table 3). Fish storage causes damage to tissue structure and destroys muscle membrane structure, which results in a decrease in muscle hardness and impedance simultaneously (Chen, et al., 2017). Overall, the relationship between impedance and rigor mortis and its resolution (distance of tail tip from the table) was relatively strong, which indicates that impedance is a useful index for fish freshness during storage. Furthermore, impedance could be used to effectively predict muscle hardness after rigor mortis.

## 4 CONCLUSION

Impedance could be used to measure fish freshness nondestructively during ice storage. Impedance measured at 2 kHz accurately detected rigor mortis and rigor‐resolution, and had a strong negative correlation with the K‐value (*r* = −0.938, *p* < .01) and rigor mortis index (*r* = −0.893, *p* < .01). Regarding hardness, a strong positive correlation was found with *r* = 0.981 (*p* < .01) after rigor mortis during storage. Therefore, impedance could be used to predict fish freshness and muscle softening. As the measurement was only carried out on rainbow trout, further research on other species should also be carried out for enhanced understanding of using impedance to predict freshness in fishes.

## CONFLICT OF INTEREST

All authors hereby declared no conflict of interest.

## ETHICAL APPROVAL

There was no human or animal testing in this study.
